# Computer-Aided Drug Design and Drug Discovery

**DOI:** 10.3390/ph18030436

**Published:** 2025-03-20

**Authors:** Dragos Paul Mihai, George Mihai Nitulescu

**Affiliations:** Faculty of Pharmacy, “Carol Davila” University of Medicine and Pharmacy, Traian Vuia 6, 020956 Bucharest, Romania; george.nitulescu@umfcd.ro

In the rapidly evolving landscape of pharmaceutical research, the integration of computational methods has become a cornerstone in drug discovery and development efforts. Computer-aided drug design (CADD) offers a more efficient and cost-effective approach, complementing traditional experimental techniques. By leveraging computational tools such as molecular modeling, structure–activity relationships, and virtual screening, researchers can predict the behavior of drug candidates, assess their interactions with biological targets, and optimize their pharmacokinetic properties before synthesis and experimental validation [1,2,3,4,5].

Over the past few decades, CADD has transitioned from a supplementary tool to a central component in drug discovery pipelines. The realm of drug discovery has been significantly transformed by the advent of CADD, which integrates computational tools with traditional pharmacological methods to streamline the discovery and development of novel therapeutic agents [6,7,8,9,10].

This Special Issue (“Computer-Aided Drug Design and Drug Discovery”) aims to showcase some of the latest advancements in this interdisciplinary field, highlighting the applications of computational approaches in accelerating the identification and optimization of therapeutic agents [11,12]. By exploiting machine learning (ML) and artificial intelligence (AI) algorithms [7], this issue seeks to contribute to the development of cutting-edge methodologies for drug design and discovery [10].

Despite the rapid evolution of CADD and its integration into modern drug discovery, several key limitations and challenges remain unaddressed. These gaps highlight the need for further advancements to optimize the efficiency, reliability, and applicability of computational drug design methodologies.

One of the biggest challenges in CADD is the availability and quality of biological and chemical datasets. Many datasets used for training AI models in drug discovery are proprietary, incomplete, or biased toward well-studied compounds, leading to reduced predictive accuracy [13]. Additionally, integrating large-scale biological data such as genomics, proteomics, and metabolomics (multi-omics integration) remains a significant challenge due to standardization issues [14]. Existing computational frameworks struggle to effectively incorporate these data types into drug design pipelines. Moreover, the lack of standardized bioinformatics tools for integrating diverse datasets hinders the development of precision medicine approaches [15]. 

While AI and ML have transformed CADD, many predictive models suffer from overfitting, lack of interpretability, and insufficient generalizability across different chemical spaces [2]. These limitations often lead to inaccurate predictions of molecular binding affinities and pharmacokinetic properties [15].

Despite technological advancements, high-performance computing (HPC) and quantum computing remain underutilized in CADD due to cost constraints and limited accessibility [13]. Many small research institutions and startups lack access to the computational resources needed for large-scale molecular simulations [14].

The use of AI in drug discovery also raises ethical and regulatory concerns, including bias in AI models, reproducibility of results, and the validation of AI-predicted drug candidates in clinical trials [16]. Additionally, there is a lack of clear regulatory frameworks for AI-driven drug discovery approaches [17].

This Special Issue directly tackles many of the key gaps in knowledge identified in CADD. The published papers showcase advancements in predictive modeling, drug repurposing, molecular docking, and machine learning-driven discovery, demonstrating how computational methodologies can overcome existing limitations in data availability, predictive accuracy, and multi-target drug discovery.

A comprehensive review by Niazi and Mariam (Contribution 1) traces the historical evolution of CADD, delineates its methodologies into structure-based and ligand-based approaches, and discusses its pivotal role in modern drug discovery. The paper also addresses current challenges, including the integration of AI and ML, data privacy concerns, and the need for robust ethical frameworks. Another review (Contribution 2) highlights the successful application of computational approaches in identifying novel SIRT1/2 modulators, providing a detailed analysis of ligand and structure-based methods employed in recent discoveries.

Several studies in this Special Issue have introduced novel methodologies to enhance model reliability. One study illustrates the development of an advanced ML-based framework for epitope selection in vaccine design, addressing the challenge of poor predictive accuracy in peptide vaccine development. By employing random forest classification and linear regression models, this study demonstrates how improved algorithms can optimize epitope safety and efficacy while ensuring population-wide vaccine applicability (Contribution 4) [18]. Another study showcases how QSAR modeling, molecular docking, and molecular dynamics simulations can be combined to repurpose existing drugs for new therapeutic uses (Contribution 21) [19].

Traditional drug discovery often fails to address multi-domain proteins or diseases with complex pathologies. One study (Contribution 5) highlighted the virtual screening of inhibitors that simultaneously bind multiple domains within protein tyrosine kinase 6 (PTK6), potentially improving inhibitor efficacy and specificity. Another study addresses drug resistance issues in acute myeloid leukemia (AML) by using molecular docking and molecular dynamics simulations to identify inhibitors that target a mutant isocitrate dehydrogenase 1 (mIDH1) variant (Contribution 10), demonstrating how CADD can lead to the discovery of second-generation inhibitors to counteract resistance mutations.

The reliability of structural modeling remains a significant challenge in CADD, particularly in homology modeling and deep-learning-based structure predictions. One paper featured in this Issue critically evaluates these methodologies and provides insights into their limitations. Homology modeling was assessed against deep learning-based AlphaFold 3D structure predictions [20]. The study identified cases where AlphaFold fails to match experimental data, emphasizing the need for hybrid computational-experimental validation approaches.

The COVID-19 pandemic has highlighted the need for rapid antiviral drug discovery, yet computational screening for antiviral compounds remains an underexplored area. One paper in this Issue addresses how CADD can accelerate the identification of antiviral agents by using molecular docking, network pharmacology, and bioinformatic tools to explore natural product-based antiviral mechanisms against SARS-CoV-2 (Contribution 3).

Another work describes the design, synthesis, and activity assessment of antibacterial and antifungal hybrid compounds combining thienopyrimidine and sulfonamide scaffolds (Contribution 7). In silico screening against target enzymes guided the selection of promising candidates, which were subsequently validated through experimental assays.

Collectively, the papers published in this Special Issue underscore the transformative impact of computational approaches in drug discovery. The integration of AI and machine learning further enhances these capabilities, enabling the analysis of complex datasets and the generation of predictive models that inform decision-making in drug development.

Despite the presented advancements, several challenges remain unresolved in the field of CADD. Future research should focus on significantly improving the accuracy of predictive models, addressing biases in AI algorithms, and ensuring the integration of diverse biological data.

## Data Availability

Data are contained in this article.
